# Transcriptome-Wide Identification of Dark- and Salt-Induced Senescence-Related *NAC* Gene Family Members in Alfalfa

**DOI:** 10.3390/ijms25168908

**Published:** 2024-08-15

**Authors:** Xiangxue Duan, Daicai Tian, Peiran Gao, Yue Sun, Xiaojing Peng, Jiangqi Wen, Hongli Xie, Zeng-Yu Wang, Maofeng Chai

**Affiliations:** 1 Key Laboratory of National Forestry and Grassland Administration on Grassland Resources and Ecology in the Yellow River Delta, College of Grassland Science, Qingdao Agricultural University, Qingdao 266109, China; 2 Institute for Agricultural Biosciences, Oklahoma State University, Ardmore, OK 73401, USA

**Keywords:** dark stress, salt stress, leaf senescence, alfalfa, expression profile

## Abstract

Leaves are a key forage part for livestock, and the aging of leaves affects forage biomass and quality. Preventing or delaying premature leaf senescence leads to an increase in pasture biomass accumulation and an improvement in alfalfa quality. NAC transcription factors have been reported to affect plant growth and abiotic stress responses. In this study, 48 *NAC* genes potentially associated with leaf senescence were identified in alfalfa under dark or salt stress conditions. A phylogenetic analysis divided *MsNACs* into six subgroups based on similar gene structure and conserved motif. These *MsNACs* were unevenly distributed in 26 alfalfa chromosomes. The results of the collinearity analysis show that all of the *MsNACs* were involved in gene duplication. Some *cis*-acting elements related to hormones and stress were screened in the 2-kb promoter regions of *MsNACs*. Nine of the *MsNAC* genes were subjected to qRT-PCR to quantify their expression and *Agrobacterium*-mediated transient expression to verify their functions. The results indicate that *Ms.gene031485, Ms.gene032313, Ms.gene08494,* and *Ms.gene77666* might be key *NAC* genes involved in alfalfa leaf senescence. Our findings extend the understanding of the regulatory function of *MsNACs* in leaf senescence.

## 1. Introduction

Leaves are the main photosynthetic organs of plants and are thus essential to growth and development [1,2]. Senescence is the final stage of plant growth and can occur in different cells, tissues, and organs at different times [3]. With leaf aging, chloroplasts begin to degenerate, accompanied by the decomposition of macromolecules, including nucleic acids, proteins, and lipids [4]. Nutrients are transferred from senescing leaves to newly developing or storage tissues, such as new shoots, leaves, flowers, and seeds [5]. Leaf senescence is controlled by leaf age and is affected by various environmental factors closely related to plant growth, development, adaptation, and reproduction [6], such as biotic and abiotic stresses [7]. Therefore, leaf senescence is a comprehensive response to leaf development over time and other internal and external stress stimuli [8,9,10].

Leaf senescence is determined by the synergistic effects of senescence regulatory genes [11]. Multiple transcription factors that mediate the regulation of leaf senescence have been identified, emphasizing the role of transcription factor-mediated transcriptional control [12]. The literature indicates that plants contain a series of transcription factors that induce senescence [13]. Among these transcription factors, NAC, WRKY, and MYB have been confirmed to be involved in plant leaf senescence [14,15,16,17].

NAC is one of the most abundant transcription factor families in plants [18]. The name NAC is derived from the symbols for the other three transcription factors: NO APICAL MERISTEM (NAM), *ARABIDOPSIS* TRANSCRIPTION ACTIVATION FACTOR (ATAF), and CUP-SHAPED COTYLEDON (CUC) [19,20]. NAC transcription factors are involved in leaf senescence mainly by regulating chlorophyll degradation genes [21]. For example, the tomato NAC transcription factor SlNAP2 [22], rice NAC transcription factor OsNAC2 [23], and corn NAC transcription factor ZmNAC132 [24] can bind to the promoter regions of chlorophyll degradation-related genes to regulate their expression. NAC transcription factors are also involved in regulating the expression of plant hormone synthesis and degradation genes. For example, the Chinese cabbage NAC transcription inhibitor BrNAC041 [25] can bind to the promoters of the ABA catabolism gene *BrCYP707A3* and the GA biosynthesis genes *BrKAO2* and *BrGA20ox2* to inhibit ABA decomposition. The GA metabolic and biosynthetic genes are linked to leaf senescence as well [26]. NAC transcription factors also participate in regulating the expression of genes related to ROS production in plants. For example, the membrane-bound transcription activator BnaNTL1 in rapeseed directly interacts with genes involved in ROS production (*RbohD*), programmed cell death (*VPEs* and *CEP1*), and leaf senescence (*BFN1*) and promotes rapeseed leaf senescence [27].

Alfalfa (*Medicago sativa* L.) is a high-quality leguminous forage with good palatability and high nutritional value [28]. However, leaf premature senescence affects the nutritional value and quality of alfalfa [29]. Therefore, preventing or delaying premature senescence to increase biomass accumulation would improve the quality and economic benefits of alfalfa [30]. To this end, in this study, we investigated the transcriptome of alfalfa leaves experiencing senescence induced by darkness and salt stress to discover unknown candidate genes that regulate leaf senescence. Our research results provide new ideas and new directions for the functional identification of leaf senescence-related genes and highlight research directions for increasing forage biomass accumulation under dense planting and salt stress. Finally, we identified genetic resources for cultivating new varieties of salt-resistant and anti-aging plants.

## 2. Results

### 2.1. Phenotypic Changes in Leaf Senescence Under Dark and Salt Stress

**Figure 1:**
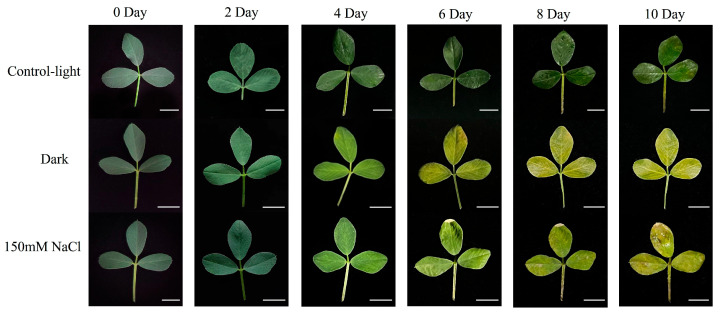
Identification of leaf senescence phenotypes in alfalfa under dark- and salt-stress-induced conditions. Senescence process of detached alfalfa leaves treated with CK (light-control), dark, and 150 mM NaCl for 0, 2, 4, 6, 8, and 10 days. Scale bar, 1 cm.

Dark-induced leaf senescence is similar to age-dependent leaf senescence during normal plant development [31]. Our previous study confirmed that the leaf senescence phenotype of the model plant *Medicago truncatula* treated with 150 mM NaCl was similar to that under dark treatment [32]; thus, we used 150 mM NaCl to treat alfalfa leaves for salt stress. We established a light-control group (CK) to eliminate the background effects of the development process. The detached leaves were treated with light (i.e., the CK), darkness, and 150 mM NaCl (Figure 1). Consistent with our previously published results in *Medicago truncatula*, the experimental results in alfalfa show that leaf senescence induced by the 150 mM NaCl treatment was similar to that induced by the dark treatment. In the salt-treated group, the leaves showed a slight whole-leaf yellowing: on day 4, the leaves began to turn yellow; on day 6, leaf yellowing was intensified; on day 8, 90% of the leaves turned yellow; on day 10, the leaves were completely yellow and senescent. In the dark-treated group, the leaves began to show a yellowing phenotype on day 4, gradually turning yellow on days 6 and 8, and completely turned yellow on day 10, showing an evident aging phenotype. However, in the CK, the leaves remained green during the first four days of treatment and no evident signs of yellowing were observed until day 10.

### 2.2. Screening NAC Genes Related to Leaf Senescence

**Figure 2:**
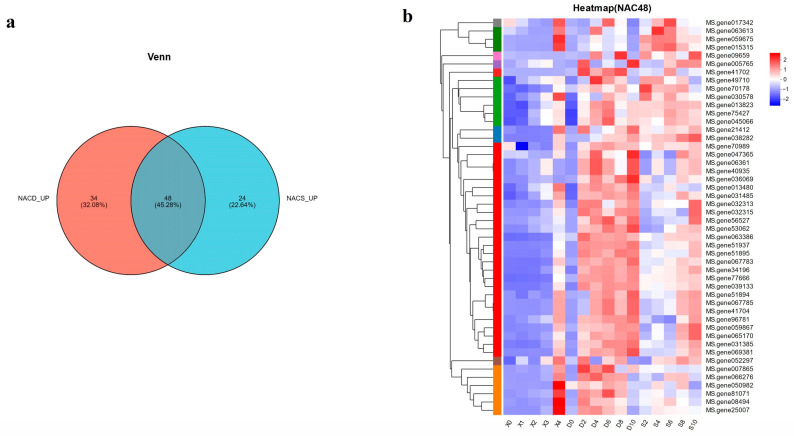
Screening of 48 aging-related *MsNACs* under dark and 150 mM NaCl treatment. (**a**) Venn diagram of *MsNAC* genes under dark (Orange) and 150 mM NaCl treatment (Blue). (**b**) Heatmap of expression levels for the 48 screened *MsNAC* genes. The horizontal row represents the gene, while the vertical row represents the treatment period. The far left of the heat map is a dendrogram of *MsNACs*, and different color bars represent different subclusters. The color bar on the right side of the heat map indicates the gene’s expression level after standardized treatment. Red indicates a high expression level of the gene, while blue represents a low expression level. The gradient from blue to red signifies a change from low to high expression.

Both dark and salt stress can cause signs of senescence in plant leaves, and the genes induced by both treatments are probably related to leaf senescence. We drew a Venn diagram of alfalfa *NAC* gene family members in the transcriptome that were induced by both dark and salt stress (Figure 2a). The results showed that 48 *MsNACs* were simultaneously induced by dark and salt stress associated with leaf senescence. Subsequently, we analyzed the expression of the genes that encoded these 48 aging−related transcription factors under natural aging (X), dark stress (D), and salt stress (S) and drew an expression heat map (Figure 2b). The expression of most of these genes was significantly higher at 2, 4, 6, 8, and 10 d after dark and salt treatments than that in the untreated period. Under dark conditions, the expression of most genes increased with the extension of treatment time. Similarly, the expression of most genes was increased with prolonged salt treatment, but the degree of upregulation was less than that under dark conditions. In the natural aging process, the transcript levels of most of these 48 *MsNACs* are also induced. X0 represents the youngest leaf blade at the top, and as development time extends, the X3 period represents the mature leaf blade, with the X4 period representing the aged leaf blade. According to the expression levels shown in Table S1, the majority of genes show an increase in expression along with leaf development time, which reached its peak during the aging stage (X4).

### 2.3. Phylogenetic Analysis

**Figure 3:**
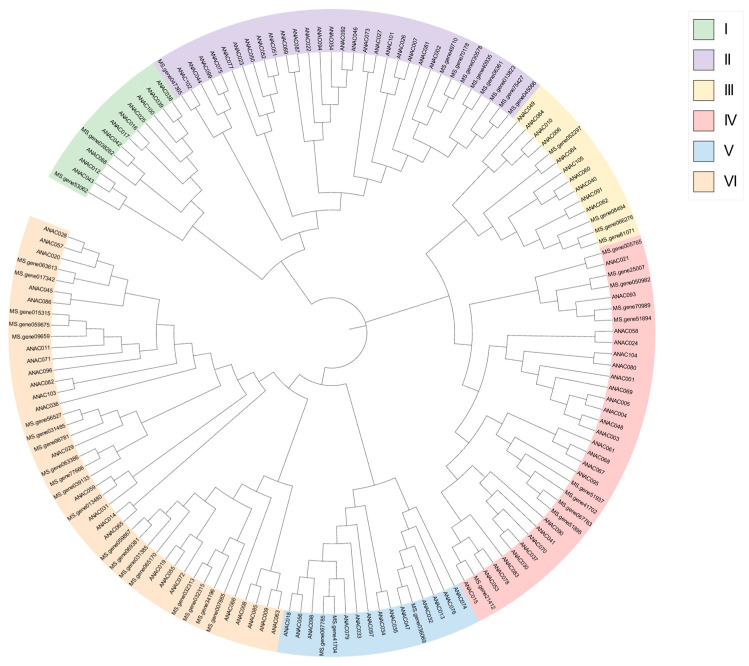
A phylogenetic analysis of *NACs* from *Medicago sativa* and *Arabidopsis thaliana* (At) was conducted. Subgroups are marked with different colors, and the subgroups’ (I–VI) color is marked in the upper right corner.

To structurally classify the 48 age-related *MsNAC* genes screened from the transcriptome, we used MEGA11.0 software to compare the 48 MsNAC proteins with the 105 AtNAC proteins in *Arabidopsis* according to their homology to construct a phylogenetic tree (Figure 3). These proteins were clustered into six groups (I–VI), and 48 MsNAC proteins were distributed throughout the six groups, but their distributions were uneven. Group I contained the fewest NAC proteins and only two MsNAC proteins. Group VI contained the most AtNAC and MsNAC proteins. *ANAC059* can induce leaf senescence [15]. *Ms.gene013480* had a very close genetic relationship with this gene, indicating that *Ms.gene013480* might also respond to alfalfa leaf senescence. *ANAC019, ANAC055*, and *ANAC072* are also positive regulators of leaf senescence [33]. *Ms.gene059867, Ms.gene069381, Ms.gene031385*, and *Ms.gene065170* were clustered into the same group as the senescence-related genes. We speculated that these *MsNACs* might also be involved in alfalfa leaf senescence. ANAC047 is a salinity-induced transcription factor and is involved in salt stress-induced senescence. Transgenic lines expressing the chimeric inhibitor ANAC047-SRDX showed significantly improved salt tolerance compared with that of control plants, indicating that ANAC047 plays a positive role in stress-induced senescence [34]. According to the evolutionary tree, *Ms.gene036069* and ANAC047 were on the same branch and had a very close genetic relationship; therefore, we speculated that *Ms.gene036069* might also positively regulate alfalfa leaf senescence.

### 2.4. Sequence and Structural Analysis of MsNACs

**Figure 4:**
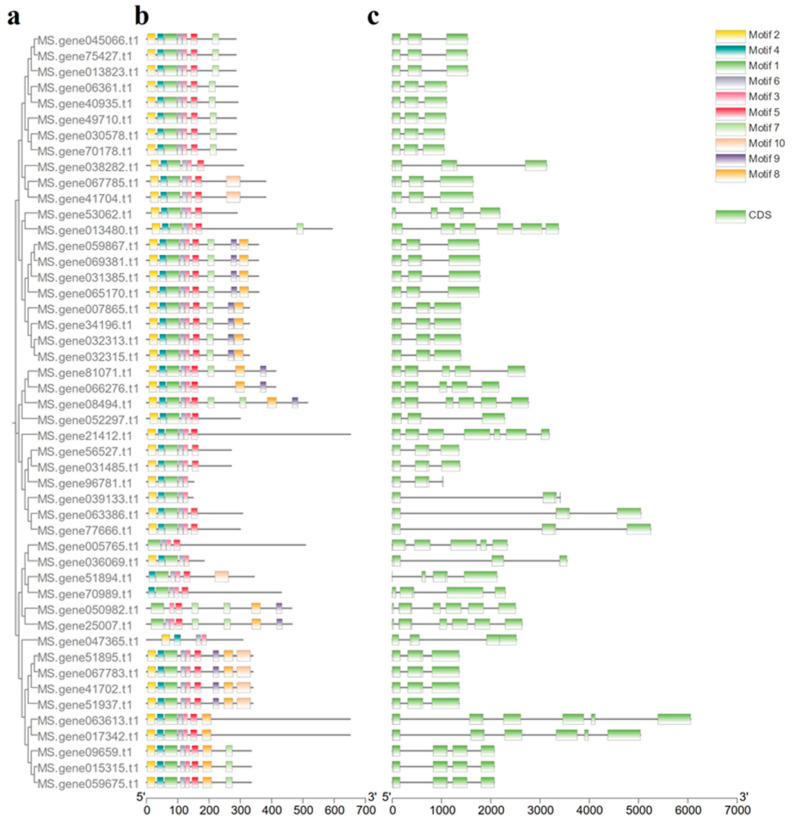
Phylogenetic relationships and motifs of *NAC* genes from *Medico sativa*. (**a**) Phylogenetic tree of 48 *MsNACs*. (**b**) Analysis of conserved elements of 48 *MsNACs*. Boxes of different colors represent different motifs. (**c**) Exon–intron organizations of 48 *MsNACs*. Green boxes indicate exons; black lines indicate introns.

We constructed a phylogenetic tree of 48 aging-related *MsNACs* and analyzed the motif features and gene structures of these 48 *MsNAC*s (Figure 4). *MsNAC* genes had high homology and a similar motif distribution. We used the MEME online program to analyze the ten conserved motifs. The number of motifs contained in the *MsNACs* ranged from four to nine. Motif 3 was found in all of the *MsNACs*, indicating its importance. Most genes contained motif 6, except for *Ms.gene050982*. Two *MsNACs* (*Ms.gene047365* and *Ms.gene005765*) each contained four motifs, which was the lowest number of motifs observed. Although *MsNACs* in different clades differed in the type and number of motifs, most *MsNACs* in the same clade had similar motifs. Gene structure analysis showed that the 48 *MsNACs* contained from two to six introns. The number and distribution of exons and introns in genes closely distributed on the phylogenetic tree was approximately the same. We found that the higher the homology of *MsNACs*, the more similar the composition and distribution of their protein motifs.

### 2.5. Chromosomal Localization and Collinearity Analysis

**Figure 5:**
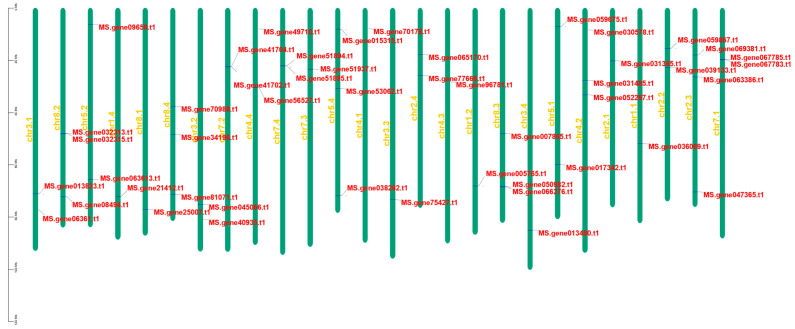
Localization analysis of 48 *MsNACs* on chromosomes. Green bars represent chromosomes; red font represents genes’ ID.

To determine the specific chromosomal locations of *MsNACs*, we drew a chromosomal distribution map of 48 aging-related *MsNAC* genes (Figure 5). The results show that 48 *MsNACs* were distributed on 26 chromosomes of alfalfa (2n = 4x = 32). chr2.3, chr4.2, chr5.4, chr8.2, chr8.3, and chr8.4 each contained the most (three) *MsNAC* genes. Only one *MsNAC* gene was found on ten chromosomes. For chromosomes 2, 3, 4, 7, and 8, *MsNAC* was present in each chromosome allele. On chromosome 6, *MsNAC* was not identified in any chromosome allele.

**Figure 6:**
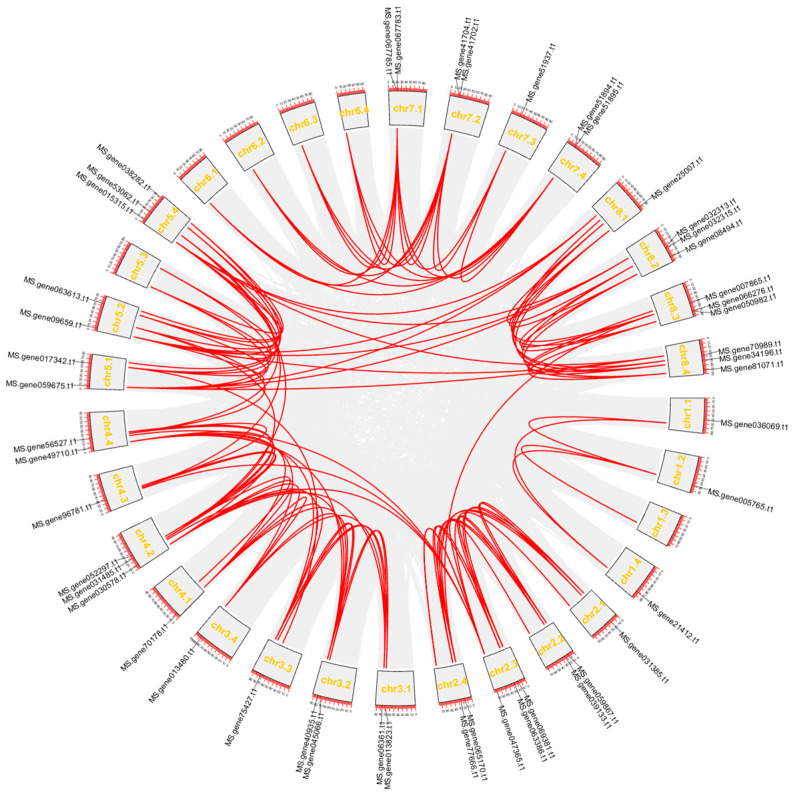
Collinear loop diagram of the interior of 48 *MsNACs.* Red lines indicate duplicated *MsNAC* gene pairs.

Gene duplication provides species with the evolutionary potential to generate new functions and is the source of genetic and evolutionary innovation. To analyze the gene duplication relationship of 48 age-related *MsNACs*, we used TBtools to perform a collinearity analysis (Figure 6). All *MsNACs* participated in gene duplication, including tandem duplication, whole-genome duplication, and chromosome segment duplication [35]. Sixteen sets of tandem duplications were found in *MsNACs*. 

### 2.6. Cis-Acting Element Analysis of MsNACs

**Figure 7:**
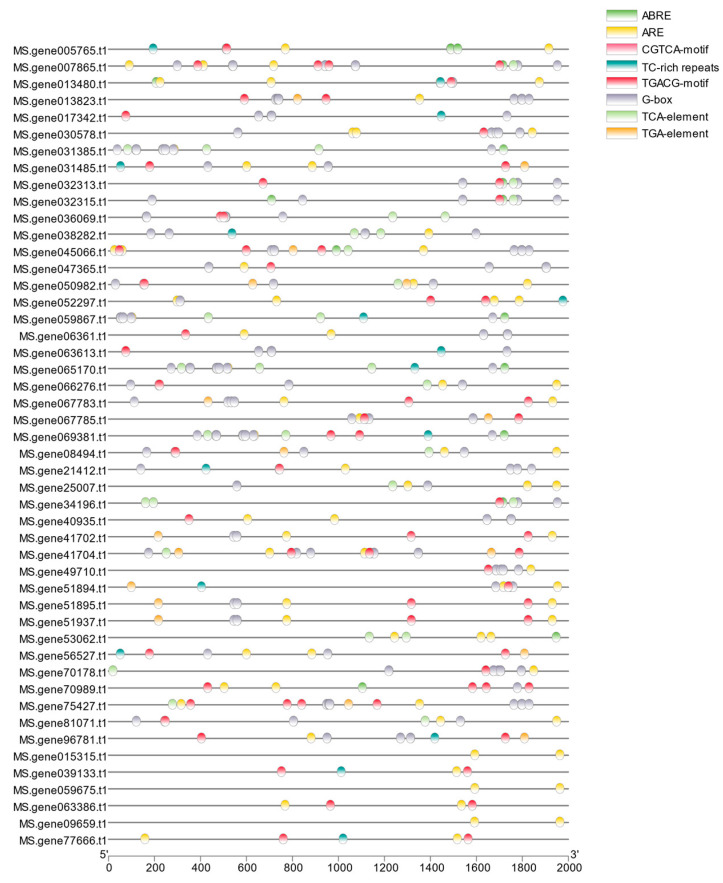
Analysis of cis-acting elements in the *MsNACs* promoter. Different cis-acting elements are represented by circles of different colors.

The promoter sequence 2000 bp upstream of the CDS region of MsNACs from the alfalfa genome were extracted, and the *cis*-acting elements of 48 *MsNACs* were predicted using PlantCARE (Figure 7). The analysis of the prediction results found that these *cis*-acting elements comprised hormone response elements and abiotic stress response elements. Among them, 42 *MsNACs* contain abscisic acid-responsive elements (ABRE), 39 *MsNACs* contain methyl jasmonate acid-responsive elements (CGTCA motif and TGACG motif), 20 *MsNACs* contain salicylic acid-responsive *cis*-acting elements (TCA-elements), and most *MsNACs* contain light-responsive *cis*-acting elements (G-box).

### 2.7. Verification of Screened Aging-Related NAC Genes

**Figure 8:**
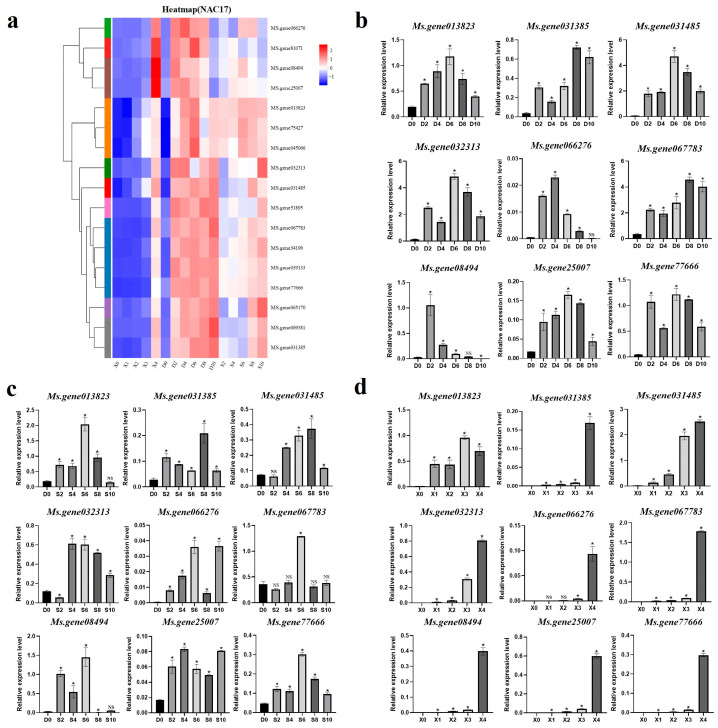
Selected *MsNACs* based on response to leaf senescence on days 4 and 6. (**a**) Heatmap of the 17 *MsNACs* identified on days 4 and 6 of dark- and salt-induced leaf senescence. The horizontal row represents the gene, while the vertical row represents the treatment period. The far left of the heat map is a dendrogram of *MsNACs*, and different color bars represent different subclusters. The color bar on the right side of the heat map indicates the gene’s expression level after standardized treatment. Red indicates a high expression level of the gene, while blue represents a low expression level. The gradient from blue to red signifies a change from low to high expression. The qRT-PCR verification of Nine *MsNACs* expression under dark stress (**b**), salt stress (**c**) and natural aging (**d**). Data in (**b**–**d**) represent mean values (±SD; n = 3) and were analyzed using Student’s *t*-test (NS; not significant, * *p* < 0.05) against D0 or X0.

From above experiments, we found that alfalfa leaves began to turn yellow on day 4 under the dark- and salt-induced treatments and senesced on day 6. Therefore, we selected *NAC* genes that were subjected to both dark and salt treatment on days 4 and 6 in the transcriptome for further analysis. Seventeen age-responsive genes were identified, and their expression patterns were analyzed for natural aging, dark stress, and salt stress, and an expression heat map was constructed (Figure 8a). The expression of these genes was increased with treatment time under both dark and salt conditions. The expression of these genes was also increased under natural aging conditions. To further verify these results, we removed the redundancies of homologous gene duplication and selected nine genes for qRT-PCR verification (Figure 8b–d). The results showed that the nine genes had higher expression levels on days 2, 4, 6, 8, and 10 of dark-induced and salt-induced treatments compared to that of the CK. During the natural senescence process, the qRT-PCR results showed the expression levels of nine genes increased with the extension of the leaf development period, and reached the maximum expression level at X4. In general, the qRT-PCR results demonstrated consistent trends shown by transcriptome data.

### 2.8. Verification of Gene Function through Transient Expression Mediated by Agrobacterium

**Figure 9:**
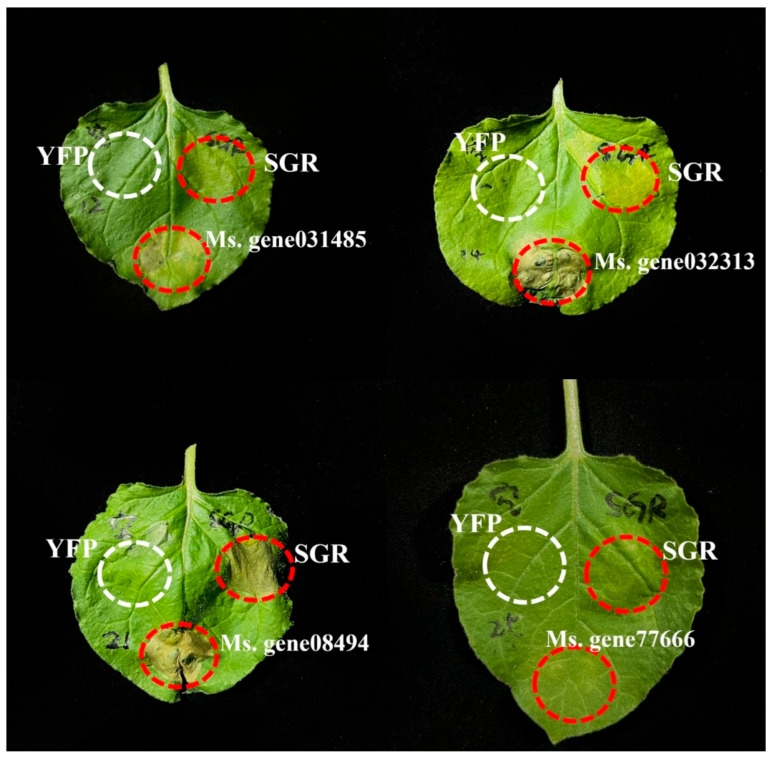
Functional validation of selected *MsNACs* using an *Agrobacterium*-mediated transient expression assay. Positive control: *SGR*. Negative control: empty vector with YFP.

To determine whether the nine selected candidate genes were truly involved in senescence, we used *Agrobacterium*-mediated transient gene expression in tobacco leaves for function verification. *MsSGR* plays important roles in the process of chlorophyll degradation during leaf senescence. [36]. We used alfalfa leaf cDNA as a template to amplify the nine senescence-responsive genes and CDS of *MsSGR* and then ligated them to the gene overexpression vector pFGC-eYFP. The ten constructed vectors and the empty vector were transformed into *Agrobacterium*-competent cells for transient overexpression in *Nicotiana benthamiana*. An empty vector was used as a negative control, and *MsSGR* was used as a positive control. The phenotypes were observed 10 h after infiltration (Figure 9). The *N. benthamiana* leaves infiltrated with *Ms.gene031485, Ms.gene032313, Ms.gene08494,* and *Ms.gene77666* overexpression vectors turned yellow to varying degrees, and the *N. benthamiana* leaves infiltrated with *MsSGR*-overexpression vector also turned yellow. The *N. benthamiana* leaves infiltrated with the empty vector remained green. Experimental results showed that heterologous overexpression of the alfalfa *NAC* genes *Ms.gene031485, Ms.gene032313, Ms.gene08494,* and *Ms.gene77666* in *N. benthamiana* leaves promoted leaf senescence. Thus, these four genes might regulate leaf senescence in alfalfa.

## 3. Discussion

In this study, we subjected leaves of alfalfa cultivar ‘Xinjiang Daye’ to darkness and salt stress treatments and subsequently performed transcriptome sequencing. Based on the leaf senescence phenotype, we screened 48 *NAC* gene family members. Our analysis showed that these genes were also induced to express during the natural aging process. We therefore considered these genes to be alfalfa aging-related genes and performed bioinformatics analysis on these 48 genes. To determine the evolutionary relationship among these 48 aging-related *NAC* genes, we compared the homology of these 48 MsNAC proteins with 105 AtNAC proteins in *Arabidopsis thaliana*, constructed a phylogenetic tree, and analyzed the gene structures and the conserved motifs. Chromosome localization and collinearity analysis of these 48 *MsNACs* were performed, and the duplicate evolutionary relationships of these genes were observed. The *cis*-acting elements of 48 aging-related *MsNACs* were predicted. qRT-PCR was used to verify the expression of the selected aging-related *NAC* genes, and these genes were cloned and infiltrated into *N. benthamiana* leaves to observe the aging phenotype.

We obtained phenotypes related to dark-induced and salt-stress-induced leaf senescence in alfalfa. In both the salt and the dark treatment groups, the leaves began to show signs of senescence on day 4, and as time passed, these signs became increasingly evident until the leaves were completely yellow and senescent on day 10. However, the light control group showed only slight color changes and remained green until day 10. Abiotic stress can accelerate leaf senescence [37]; thus, we drew a Venn diagram of the alfalfa *NAC* genes induced by both dark and salt stress in the transcriptome. Forty-eight *NAC* genes that may affect alfalfa leaf senescence were identified, and the expression levels of these genes under stress induction were analyzed. We found that the expression of these genes increased as treatment time increased. Under natural aging conditions, the expression of the 48 aging-related candidate genes also increased with time.

According to the phylogenetic relationship of genes, 48 aging-related *NAC* genes were divided into six subgroups. *ANAC059, ANAC019, ANAC055, ANAC072,* and *ANAC047* are involved in leaf senescence in *Arabidopsis thaliana* [21,34]. Therefore, based on the established phylogenetic tree, the functions of *MsNACs* in the same subgroup could be inferred. It was preliminarily inferred that *MsNACs* in the same subgroup as the aforementioned genes may be involved in alfalfa leaf senescence. The structure of genes reflects the genetic evolution of different genes in the same species. We analyzed the structure and conserved base order of 48 aging-related *MsNACs* and confirmed the phylogenetic classification by the distribution of introns and motifs. The *MsNACs* with close proximity had similar intron structures and positions, and these genes may have similar functions. 

The 48 *MsNACs* are unevenly distributed on 26 of the 32 alfalfa chromosomes, except for chromosome 6. Gene duplication is the driving force of inheritance and genetic simplification, and gene replication is the basis for the generation of new genes [38]. NAC transcription factors in different plants share a common ancestor and undergo duplication events [39]. Most species have segmental duplications and tandem repeats as the dominant events [40]. In other species, such as *Panicum miliaceum*, *Musa acuminata*, and peanuts, duplication events in the *NAC* gene family have also been reported [41,42,43]. The results of collinearity analysis showed that *MsNACs* were mainly replicated in tandem.

The *cis*-acting elements in the gene promoter region indicate that the gene may play different biological functions under different stresses [44]. In this study, we found that the promoters of the 48 *MsNACs* mainly included hormone response elements and abiotic stress response elements. According to the prediction of *cis*-acting elements, the abscisic acid-responsive element motif was the most extensively present motif among the 48 *MsNACs*, which also contained many CGTCA motifs and TGACG motifs related to MeJA responsiveness. MeJA responsiveness is related to plant defense mechanisms [45]. In addition to the essential *cis*-acting elements, salicylic acid-related TCA elements and auxin-response-related TGA elements were found in the promoter regions of these genes, suggesting that they play essential roles in regulating the development and stress responses in alfalfa.

Existing phenotypic experiments indicate that alfalfa begins to turn yellow and senesce on day 4 of dark and salt treatment and transformed into a yellow-green state on day 6. Therefore, we screened the transcriptome for genes that were simultaneously affected on days 4 and 6. These 17 *MsNACs* were speculated to be age-related genes. We analyzed the expression patterns and expression levels of these 17 genes. After removing homologous genes, nine genes were selected for qRT-PCR validation. The qRT-PCR results showed that the expression levels of these nine genes were significantly higher than those of CK after dark treatment and salt treatment. Under natural aging conditions, the expression of these aging-related genes also increased with time. The aforementioned results are consistent with the trend observed in the transcriptome data. Subsequently, we overexpressed these nine genes in *N. benthamiana* to determine whether the nine selected genes have aging functions. Four (*Ms.gene031485, Ms.gene032313, Ms.gene08494,* and *Ms.gene77666*) of the nine genes were involved in senescence to varying degrees. We speculated that these four genes might regulate the senescence of alfalfa leaves.

Studying the regulatory mechanisms underlying leaf senescence in alfalfa is crucial for improving crop yields. In summary, these genes can serve as candidate genes for alfalfa leaf senescence, laying a foundation for subsequent research.

## 4. Materials and Methods

### 4.1. Plant Material and Treatment

We used *Medicago sativa* ‘Xinjiang Daye’ as the research plant material. Seeds were imbibed and transferred to a Petri dish with moist filter paper for germination. When the seeds grew 3-4 cm in the radicle, we transferred them to Hoagland’s nutrient solution for hydroponic cultivation. *Nicotiana benthamiana* seeds were placed on moist filter paper and planted in pots after germination. All plant materials were placed in an artificial climate chamber (16 h light/8 h dark, temperature 22 °C). They grew at a humidity of 65% and a light intensity of 150 μmol·m^−2^·s^−1^.

When the seedlings were 4 weeks old, we cut off the compound leaves of the third position from apex and placed them in a culture dish containing a solution without salt stress treatment or a solution containing 150 mM NaCl. The dishes were then placed in light (no salt stress treatment, salt stress treatment) or darkness (no salt stress treatment), conditions identical to those under which the plants were grown hydroponically. The NaCl -free stress treatment solution was prepared by adding 2.4 g Murashige–Skoog medium and 3 mM MES buffer to 1 L ddH_2_O, and had a pH of 5.8, and the salt stress solution was supplemented with 150 mM NaCl.

Treated leaves at different times (0, 2, 4, 6, 8, and 10 d) were collected for subsequent transcriptome sequencing and qRT-PCR verification. Three biological replicates were set up.

### 4.2. RNA Isolation

The total RNA was isolated using TRIzol^®^ Reagent (Plant RNA Purification Reagent for plant tissue; Invitrogen Corp., Carlsbad, CA, USA) for subsequent transcriptome sequencing. DNase I (TaKaRa, Kusatsu, Japan) was used for genomic DNA removal. RNA concentration was measured using a 2100 Bioanalyzer (Agilent; Santa Clara, CA, USA). To construct sequencing libraries, we selected RNA samples of good quality.

### 4.3. Differential Expression Analysis

For multi-sample (≥2) projects, we performed a differential expression analysis of genes between samples. The screening of differentially expressed genes used DESeq2. The screening parameters were |log2FC| ≥ 1.585 and padjust < 0.05. When a gene met both parameters, it was considered to be a differentially expressed gene.

### 4.4. Sequence and Phylogenetic Analysis

Visualization of the gene structure and motif distribution was conducted using TBtools- II v2.096 software [46]. The phylogenetic tree was constructed by combining 105 NAC protein sequences in *Arabidopsis* and 48 senescence-related NAC protein sequences in alfalfa using MEGA 11.0 software. [47]. PlantCARE was used to predict the *cis*-acting elements in the 2000 bp upstream CDS region of 48 aging-related *MsNACs* [48]. TBtools visualized the aforementioned information.

### 4.5. Chromosome Distribution, and Collinearity Analysis

The location of the 48 *MsNACs* was obtained from genome assembly files, and gene locations on the chromosomes were mapped using TBtools. TBtools was used to perform collinearity analysis on 48 *MsNACs* and to detect duplication events between genes.

### 4.6. Quantitative Real-Time PCR Validation of Gene Expression

To verify the expression of *MsNAC*s screened from RNA-seq, we extracted RNA from the samples at each time point in RNA-seq, and qRT-PCR was performed for verification [49]. After total RNA extraction using the Takara MiniBEST Plant RNA Extraction Kit, it was reverse-transcribed into cDNA by using HiScript IV RT SuperMix for qPCR (+ gDNA wiper). qRT-PCR validation used ChamQ SYBR Color qPCR Master Mix, and the *MsUBC* gene was used as the housekeeping gene. qRT-PCR was performed using the Bio-Rad CFX96 system. Three replicates were designed for each experiment. Primers were designed using NCBI, and the primer sequences are listed in Table S2.

### 4.7. Tobacco Transient Expression to Verify Gene Function

Ten genes were selected for transient expression in *N. benthamiana*, and the positive control was the *SGR* gene. By using cDNA as a template, the cloning and sequencing of target genes were performed to verify genetic accuracy. The nine genes and *MsSGR* selected were connected to the PFGC-eYFP vector, and the plasmid was transformed into *E. coli* DH5α. The positive colonies were selected for sequencing. After the sequencing results were correct, the plasmid was transferred into GV3101 Agrobacterium-competent cells, and the monoclonal positive colonies were selected for injection into *Nicotiana benthamiana*. [50]. CE Design software (https://crm.vazyme.com/cetool/simple.html accessed on 5 August 2024) was used to design the primers (Table S3).

### 4.8. Data Processing and Analysis

qRT-PCR data were collated and analyzed using Excel 2022 software. Relative expression was calculated using the 2^−ΔCT^ method. Mean ± SD were used to express expression data, and significant differences were detected via ANOVA analysis using GraphPad Prism 8 (NS; not significant, * *p* < 0.05).

## Data Availability

The original contributions presented in the study are included in the article/Supplementary Materials, further inquiries can be directed to the corresponding author.

## Supplementary Materials

The following supporting information can be downloaded at: https://www.mdpi.com/article/10.3390/ijms25168908/s1.
